# A practice-changing culture method relying on shaking substantially increases mitochondrial energy metabolism and functionality of human liver cell lines

**DOI:** 10.1371/journal.pone.0193664

**Published:** 2018-04-19

**Authors:** Aziza A. A. Adam, Vincent A. van der Mark, Joanne M. Donkers, Manon E. Wildenberg, Ronald P. J. Oude Elferink, Robert A. F. M. Chamuleau, Ruurdtje Hoekstra

**Affiliations:** 1 Tytgat Institute for Liver and Intestinal Research, Academic Medical Center (AMC), University of Amsterdam, Amsterdam, The Netherlands; 2 Experimental Surgical Laboratory, Academic Medical Center (AMC), University of Amsterdam, Amsterdam, The Netherlands; 3 Department Of Gastroenterology and Hepatology, Academic Medical Center (AMC), University of Amsterdam, Amsterdam, The Netherlands; Ospedale Casa Sollievo della Sofferenza, ITALY

## Abstract

Practice-changing culturing techniques of hepatocytes are highly required to increase their differentiation. Previously, we found that human liver cell lines HepaRG and C3A acquire higher functionality and increased mitochondrial biogenesis when cultured in the AMC-Bioartificial liver (BAL). Dynamic medium flow (DMF) is one of the major contributors to this stimulatory effect. Recently, we found that DMF-culturing by shaking of HepaRG monolayers resulted in higher mitochondrial biogenesis. Here we further investigated the effect of DMF-culturing on energy metabolism and hepatic functionality of HepaRG and C3A monolayers. HepaRG and C3A DMF-monolayers were incubated with orbital shaking at 60 rpm during the differentiation phase, while control monolayers were maintained statically. Subsequently, energy metabolism and hepatic functionality were compared between static and DMF-cultures. DMF-culturing of HepaRG cells substantially increased hepatic differentiation; transcript levels of hepatic structural genes and hepatic transcription regulators were increased up to 15-fold (Cytochrome P450 3A4) and nuclear translocation of hepatic transcription factor CEBPα was stimulated. Accordingly, hepatic functions were positively affected, including ammonia elimination, urea production, bile acid production, and CYP3A4 activity. DMF-culturing shifted energy metabolism from aerobic glycolysis towards oxidative phosphorylation, as indicated by a decline in lactate production and glucose consumption, and an increase in oxygen consumption. Similarly, DMF-culturing increased mitochondrial energy metabolism and hepatic functionality of C3A cells. In conclusion, simple shaking of monolayer cultures substantially improves mitochondrial energy metabolism and hepatic differentiation of human liver cell lines. This practice-changing culture method may prove to prolong the *in-vitro* maintenance of primary hepatocytes and increase hepatic differentiation of stem cells.

## Introduction

Highly differentiated human hepatocytes from proliferative sources are needed to serve as predictive hepatocyte model *in vitro* and as biocomponent for Bio-Artificial Livers (BALs). However, to date, hepatocytes deriving from different proliferative sources, as stem cells, induced pluripotent stem cells and liver cell lines, are deficient in complex hepatic functions [1].

HepaRG is the human liver progenitor cell line increasingly used as human liver model for the prediction of hepatotoxicity and human liver infections, and is also applied as biocomponent in the AMC-Bio-Artificial liver (AMC-BAL), [2–4] as the functionality is relatively high, and its transcriptome closely resembles that of primary human hepatocytes [5]. HepaRG cultures develop during 28 days into a mixed heterogeneous culture with hepatocyte-islands and bile duct-like cells. Treating HepaRG cells with 2% dimethylsulfoxide (DMSO) during the last two weeks of culturing, enhances their hepatic differentiation and the detoxification properties, however, it also increases cell damage [3, 6]. Interestingly, HepaRG cells cultured in the AMC-BAL platform have increased hepatic functionality and integrity compared to HepaRG monolayer cultures [7]. Of particular interest, BAL-cultured HepaRG cells efficiently eliminate lactate, while monolayer-cultured cells produce lactate [8–10]. Lactate elimination is a hallmark function of highly differentiated hepatocytes, and is lacking in the available hepatocyte culture models. Consistent with this, we recently found that BAL-culturing enhances mitochondrial biogenesis and mitochondrial activity, resulting in a shift of energy metabolism towards oxidative phosphorylation (OxPhos) [11].The stimulatory effect of the AMC-BAL culture on mitochondrial biogenesis also applied to another human liver cell line, C3A, a sub-clone of the HepG2 hepatoma cell line [12]. Among the driving factors underlying this metabolic shift is the presence of dynamic medium flow (DMF) in the BAL system [13]. We mimicked the DMF of the BAL by placing monolayer cultures into a shaking incubator (at 60 rpm) during the differentiation phase. Culturing of HepaRG monolayers with DMF increased their mitochondrial abundance 3.3- fold and the expression levels of mitochondrial inner membrane OxPhos complexes [11]. Recent studies reported a strong, likely causal, relationship between mitochondrial energy metabolism and differentiation of stem cells. On one hand, inhibition of OxPhos augments the expression of pluripotency markers in stem cells [14, 15]. On the other hand, increased mitochondrial biogenesis with a shift in cellular energy metabolism towards Oxphos is associated with the differentiation of stem cells, *e*.*g*. into hepatocyte-like cells [16].

In the current work, we investigated whether the increased mitochondrial biogenesis in HepaRG DMF-monolayers is also positively associated with hepatic functionality, and extended the study for cell line C3A.

## Material and methods

### Cell culture

HepaRG cells were kindly provided by Biopredic International (Rennes, France). HepaRG cells and C3A cells (ATCC® CRL10741™) were maintained in HepaRG medium, as described [3, 4, 10]. For testing, the HepaRG and C3A cells were seeded in 12-well culture plates (Corning, Corning, USA), unless indicated otherwise, and cultured under either Static or DMF-regimen. The Static groups were cultured under conventional conditions, *i*.*e*. static in a humidified atmosphere of 95% air and 5% CO_2_. One group was supplemented with 2% DMSO for the last two weeks of culturing (HepaRG-DMSO-Static), and the other was maintained without DMSO (HepaRG-Static).The DMF-group was kept for an initial phase (proliferation phase) under conventional conditions, being14 days for HepaRG and 3 days for C3A cells, followed by a 14 days (HepaRG cells) or 11 days (C3A cells) shaking-phase under the same atmosphere in a shaking incubator (Eppendorf, Westbury, USA) with 60 rpm orbital-shaking frequency. Three days prior testing, the cultures were supplemented with 1 mM carbamoyl-glutamate (CAG) (Sigma-Aldrich, St. Louis, USA) to stimulate carbamoyl phosphate synthetase 1 (CPS1) activity [6]. All cultures were maintained at 37°C and were negative for mycoplasma.

### Hepatic function test

The cultures were tested for their functionality, as described [9]. Briefly, monolayer cultures were exposed to 1.5 ml test medium, based on HepaRG medium supplemented with 1 mM CAG, 1.5 mM NH_4_Cl, 2.27 mM D-galactose, 2 mM L-lactate and 125 μM testosterone (all compounds from Sigma-Aldrich). During the function test, medium samples were taken at 45 min (0.5 mL) and at 24 h (1 ml), in which L-lactate, ammonia, urea, glucose, bile acid and human albumin concentrations were measured. L-lactate determination was performed using L-lactic acid assay kit (Megazyme, Wicklow, Ireland). Ammonia was assessed using Ammonia assay kit (Megazyme). Urea production was measured according to the blood urea nitrogen protocol (Sigma). Glucose concentration was determined with a glucosemeter (Contour® next) (Bayer, Leverkusen, Germany). In addition, total bile acid concentration was determined using the DIAZYME total bile acid assay kit (DIAZYME Laboratories, Dresden, Germany). Human albumin was measured using the Human Serum Albumin DuoSet ELISA according to supplier’s instructions (R&D systems Inc., Minneapolis, USA).

Metabolic rates were determined by calculating the changes in concentration in medium in time and then normalized to the protein content per well. The cultures were also tested for baseline and induced CYP3A4 activity using the P450-Glo™ CYP3A4 (Luciferin-IPA) Assay kit (Promega, Madison, USA). CYP3A4 activity was optionally induced by a 3-day treatment with 10 μM rifampicin (Sigma-Aldrich) prior to testing. The measured CYP3A4 activity, expressed as Relative Luminescence Units (RLU), was normalized to protein content per well. Total protein concentration was determined using the Bio-Rad protein assay (Bio-Rad, Irvine, USA), according to manufacturer’s protocol.

### Oxygen consumption determination

HepaRG-Static and DMF-cultures were fully differentiated in 24-well culture plates (OxoDish®, PreSens, Regensburg, Germany) with oxygen sensor spots at the bottom of the well. Pericellular oxygen concentrations were measured through the bottom of the culture plates using the SDR SensorDish® Reader, which was kindly made available by Applikon Biotechnology, Delft, the Netherlands. The measured oxygen consumption was normalized to protein content per well.

### Quantitative reverse transcription-polymerase chain reaction (qRT-PCR)

Total RNA was isolated using the RNeasy Mini Kit (Qiagen, Hilden, Germany), and qRT-PCR was performed as described [9]. Transcript levels were normalized for 18S ribosomal RNA. Primer sequences and amplicon sizes are given in S1 Table. Transcript levels of Static and DMF- cultures of HepaRG and C3A were expressed as a % of the average of two human healthy liver samples of two female patients aged 40 and 41 years undergoing liver resection, in S2 Table and S3 Table respectively.

### Taurocholate uptake assay

Taurocholate (TC) uptake was measured as described [17], using chemicals from Merck (Darmstadt, Germany), unless indicated otherwise. The cultures were washed twice with uptake buffer (5 mM KCl, 1.1 mM K_2_HPO4, 1 mM MgCl_2_ (Sigma-Aldrich) 1.8 mM CaCl_2_, 10 mM D-Glucose, 10 mM HEPES, 136 mM NaCl, pH 7.4), and then exposed to 500 μL uptake buffer containing 10 μM ^3^H-labelled TC (PerkinElmer, Waltham, USA) for 2 min at 37°C. Subsequently, the cells were washed 4x with ice-cold PBS and lysed in 0.05% SDS for 30 min at room temperature. Radioactivity was measured by liquid scintillation counting using the TRI-CARB 2900 TR (PerkinElmer) and data was normalized to protein content per well.

### Immunofluorescence staining

Cultures in 6-well plates were fixed with 2% formalin (VWR, Radnor, USA) for 2–5 min at room temperature prior to permeabilization with 0.3% Triton-X100 (Bio-Rad) in cold PBS, for 20 min on ice. The monolayers were blocked for 1h with 10% fetal calf serum (BioWhittaker, Walkersville, USA) in PBS on ice before overnight incubation with the primary antibody diluted 1:200 in PBS at 4°C. Cultures were washed 3x with cold PBS, incubated 2h at 4°C with secondary fluorescent antibody diluted 1:1000 in PBS, then washed 3x with cold PBS before incubation with DAPI-containing Vectashield (Vector Laboratories, Burlingame, USA). Imaging was performed with a DM6000B fluorescent microscope (Leica Microsystems, Wetzlar, Germany) and images were processed using ImageJ software (http://imagej.nih.gov/ij/).

Primary antibodies: Goat anti-human albumin (Bethyl Laboratories, Montgomery, USA), Rabbit anti-human SOX9 (Millipore, Billerica, USA), Goat anti-human CEBPa (Santa-Cruz, Dallas, USA), Rabbit anti-rat OATP1a1 (Alpha Diagnostics, San Antonio, USA), Mouse anti-human MRP2 (Enzo Life Sciences, Oyster Bay, USA).

Fluorescent secondary antibodies were obtained from Invitrogen, unless indicated: Donkey anti-rabbit/Alexa Flour-546, Donkey anti-goat/Alexa Flour-448 (Molecular Probes, Eugene, USA), Donkey anti-goat/Alexa Flour-546, Goat anti-rabbit/Alex Flour-448, Goat anti-mouse/Alexa Flour-546.

### Cellular polarization assay

To assess cellular polarization, the cultures were incubated with 20 μM 5-carboxyfluorescein diacetate (CFDA; Molecular Probes, Eugene, USA) in PBS at 37°C for 15 min to allow its internalization and subsequent translocation into the canalicular lumen by the multidrug resistance protein 2 (MRP2) ATP-binding cassette transporter. Then the cultures were washed 2x with PBS and mounted with DAPI containing Vectashield. Imaging was performed with fluorescent microscope (Leica Microsystems).

### Statistical analyses

Statistical analyses were performed in Prism version 7 (GraphPad Prism Inc. San Diego, USA) using Student’s *t*-tests for the comparison between two groups.

## Results

### DMF-culturing enhances HepaRG cell differentiation

**Fig 1:**
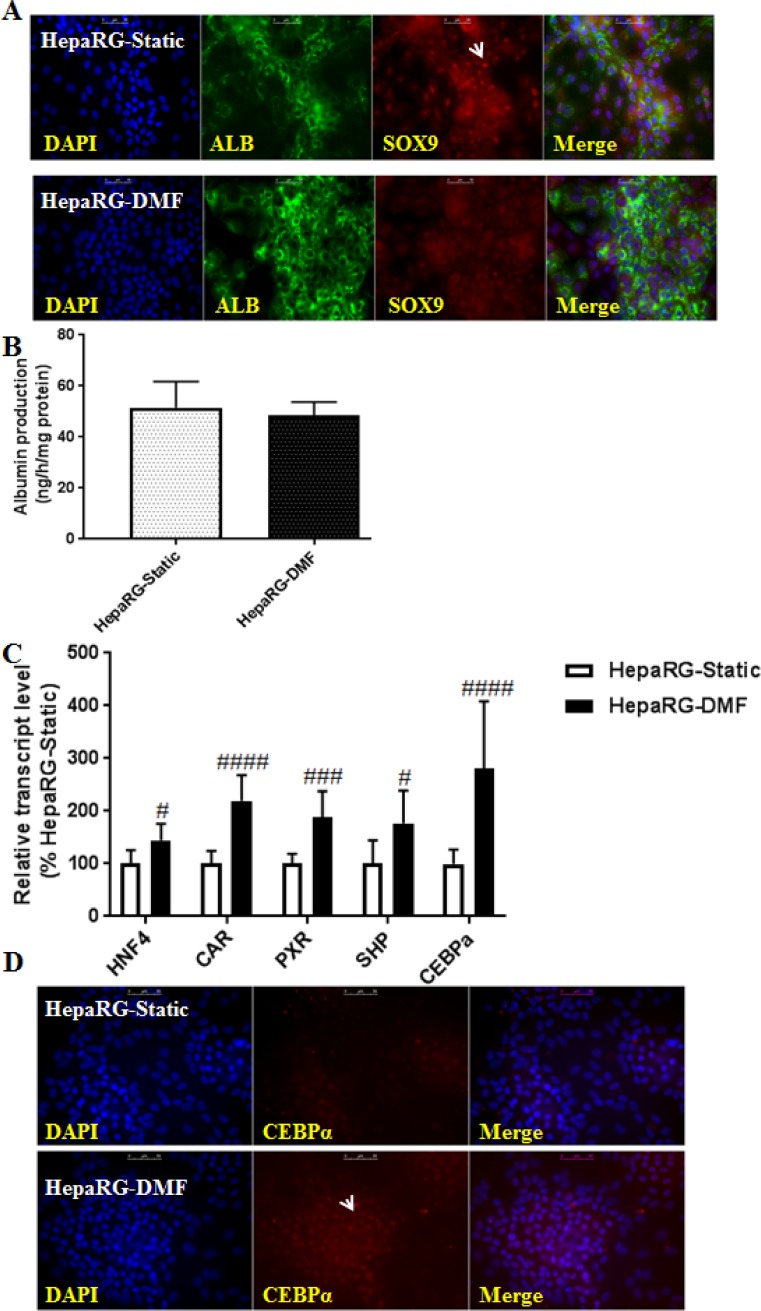
DMF-culturing enhances the differentiation of HepaRG cells. **(A)** Staining of albumin (green), and SOX9 (red), with DAPI counter-staining of the nuclei (blue) in HepaRG-Static and HepaRG-DMF. The arrow indicates the nuclear translocation of SOX9 in HepaRG-Static, scale bar = 50μm. **(B)** Albumin synthesis. **(C)** Transcript levels of genes encoding hepatic transcription regulators, n = 6-9/group. Significance Static *vs* DMF: # = *P* value <0.05, ## = *P* value <0.01, ### = *P* value <0.001 and #### = *P* value <0.0001. **(D)** Staining for CEBPα (red), with DAPI counter-staining of the nuclei (blue). The arrow indicates nuclear translocation of CEBPα, observed in HepaRG-DMF, scale bar = 50μm.

We assessed whether DMF-culturing increased the hepatic differentiation of HepaRG cells compared to conventional static culturing by co-staining of two markers; albumin (ALB), as a marker of differentiated hepatocytes, and Sry (sex determining region Y)-box 9 (SOX9), as a progenitor cell marker. SOX9 is a transcription factor, highly expressed in pluripotent, fetal, and adult stem and progenitor cells that maintains their undifferentiated-status [18, 19]. HepaRG-DMF cultures exhibited decreased SOX9 expression compared to HepaRG-Static cultures (Fig 1A and S1 Fig for higher resolution). In addition, HepaRG-Static cultures showed predominant SOX9 nuclear translocation, which was less prominent in HepaRG-DMF cultures. Furthermore, the expression of SOX9 was also studied in HepaRG-Static controls supplemented with 2% DMSO (HepaRG-DMSO-Static). Similarly, the expression of SOX9 was clearly reduced in HepaRG-DMSO-Static cultures compared to HepaRG-Static (S1 Fig). The expression of albumin was confined to hepatocyte islands and was not different between Static and DMF-cultures (Fig 1A and S1 Fig). In line with this, human albumin synthesis measured by ELISA was comparable in HepaRG-DMF and HepaRG-Static cultures and significantly higher than the level of primary human hepatocytes (PHHs) (Fig 1B, S4 Table).

Furthermore, the transcript levels of critical hepatogenic differentiation regulators [20] hepatic nuclear factor 4 (*HNF4*), constitutive active receptor (*CAR*), pregnane X receptor (*PXR*), small heterodimer partner (*SHP*) and CCAAT/enhancer-binding protein (*CEBPa*) were increased by 1.5-, 2.3-, 1.8-, 1.8- and 2.9-fold, resp. in HepaRG-DMF *vs* HepaRG-Static (Fig 1C, S2 Table). Noteworthy, the transcript levels of *HNF4*, *CAR* and *PXR* were not changed in static HepaRG cultures supplemented with 2% DMSO compared to HepaRG-Static controls [6]. The expression of CEBPα, a nuclear factor that regulates hepatic maturation and the urea cycle, was increased with more nuclear translocation in HepaRG-DMF compared to HepaRG-Static cultures (Fig 1D and, S2 Fig for higher resolution).

### DMF-culturing improves nitrogen metabolism in HepaRG cells

**Fig 2:**
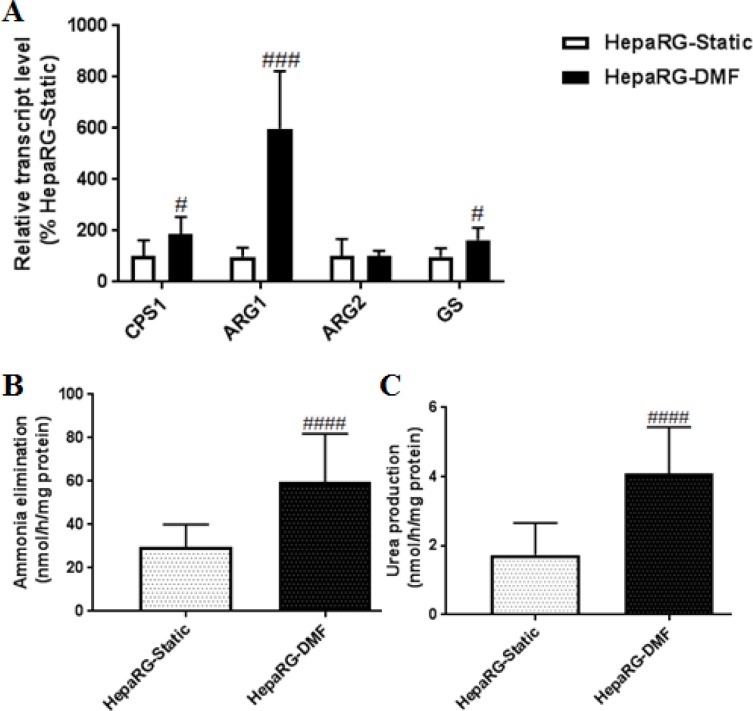
DMF-culturing improves nitrogen metabolism in HepaRG cells. **(A)** Transcript levels of genes involved in nitrogen metabolism, n = 6/group. **(B-C)** Ammonia elimination and urea production, n = 9-18/group. Significance Static *vs* DMF: # = *P* value <0.05, ## = *P* value <0.01, ### = *P* value <0.001 and #### = *P* value <0.0001.

We further assessed the effect of DMF-culturing on ammonia elimination, as one of the hallmark functions of hepatocytes. There are 2 principal routes of ammonia elimination in hepatocytes, either by irreversible conversion into urea through urea cycle activity or by reversible fixation into amino acids, primarily through the activity of glutamine synthase (GS) [21]. The urea cycle consists of 5 enzymatic reactions catalyzed by 5 enzymes with Carbamoyl Phosphate Synthetase I (CPS1) and Arginase 1 (ARG1) being two of the most critically expressed proteins in HepaRG cells [6]. ARG1 catalyzes the last step yielding urea and ornithine through hydrolysis of arginine, however, a similar reaction can be catalyzed by an extra-hepatic mitochondrial isoform, ARG2 [22, 23]. DMF-culturing induced the transcript levels of *CPS1*, *ARG1* and *GS* 1.9-, 6.0- and 1.6-fold, resp. (Fig 2A, S2 Table). Consistent with this, ammonia elimination and urea production were 2.0- and 2.4-fold increased in the DMF-cultures resp., ammonia elimination was restored to the physiological level (Fig 2B and 2C, S4 Table). As *ARG2* transcript levels remained unchanged under DMF-culturing (Fig 2A, S2 Table), it is likely that the increased urea production under DMF can be attributed to increased urea cycle activity. In DMSO-treated static monolayers, the transcript levels of *CPS1* and *GS* were 28.2- and 2.4-fold decreased compared to HepaRG-Static monolayers. However, the ureagenesis and ammonia elimination were 1.6- and 1.5-fold upregulated compared to HepaRG-Static cultures [6].

In brief, DMF-culturing improved ammonia metabolism of the HepaRG cells to higher extent compared to static cultures with or without DMSO supplementation.

### DMF-culturing substantially increases the detoxification properties and bile acid production of HepaRG cells

**Fig 3:**
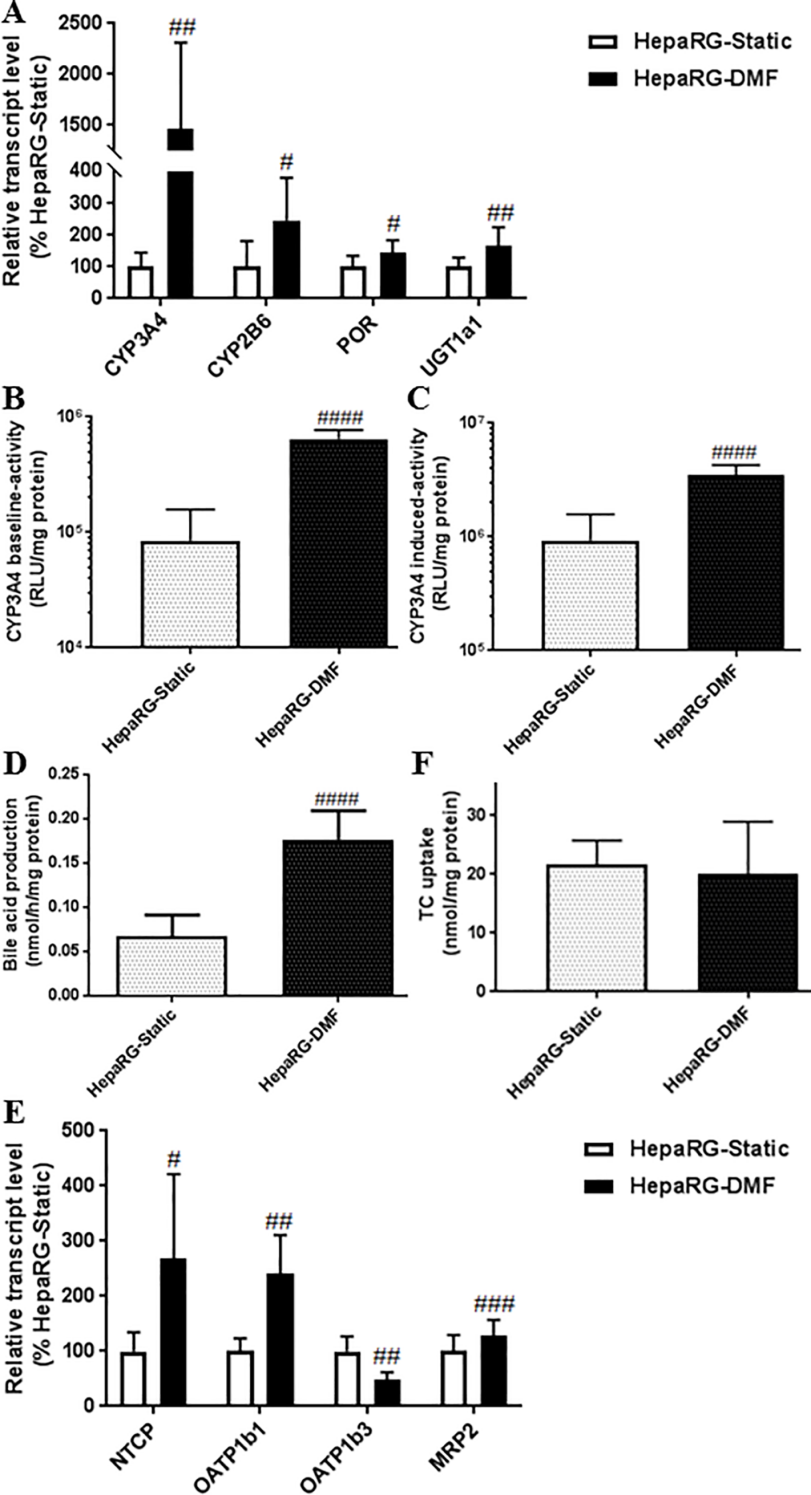
DMF increases detoxification properties of HepaRG cells. **(A)** Transcript levels of detoxification genes, n = 6-9/group. **(B-C)** CYP3A4 baseline- and induced-activity, n = 6-15/group. **(D)** Bile acid production, n = 6-15/group. **(E)** Transcript levels of hepatic transporter genes, n = 6-9/group. (F) TC uptake, n = 6/group. Significance Static *vs* DMF: # = *P* value <0.05, ## = *P* value <0.01, ### = *P* value <0.001 and #### = *P* value <0.0001.

DMF-culturing increased markers of detoxification at all three phases; phase 1, the alteration of the chemical structure to more water-soluble moiety, executed primarily by CYPs, phase 2 involving the conjugation of the hydrophilic moiety [24, 25], and phase 3, the transport into the canalicular space or circulation [26], as well as bile acid synthesis, which is essential for the secretion phase. The transcript level of *CYP3A4*, a CYP enzyme, involved in the detoxification process of 50% of prescribed drugs [27], was substantially upregulated up to 15-fold by DMF-culturing, whereas the transcript levels of *CYP2B6* and UDP-glucuronosyltransferase *(UGT1a1*) were 2.5- and 1.8-fold increased (Fig 3A, S2 Table). In the past, we found that DMSO-treatment increased the transcript levels of *CYP2B6* and *CYP3A4* 20.3- and 6.9-fold resp. [6]. Furthermore, the transcript level of cytochrome P450 oxidoreductase (*POR*), that donates electrons to CYPs, and is therefore essential for the biosynthesis of bile acid, as well as the metabolism of more than 80% of drugs in use [28, 29], was 1.6-fold upregulated in DMF-cultures (Fig 3A, S2 Table). The substantial induction of *CYP3A4* transcript levels was in line with a 7.6- and 3.8-fold increase in the baseline and induced CYP3A4 activity (Fig 3B and 3C). Bile acid synthesis was 2.6-fold augmented in the DMF-cultures (Fig 3D, S4 Table). The transcript levels of hepatic uptake transporters Na+-taurocholate co-transporter poly peptide (*NTCP*) and organic anion transporter poly peptide (*OATP1b1*) were 2.7- and 2.3- fold induced under DMF-culturing, reaching 36.7% and 6.4% of human liver level, resp. (Fig 3E, S2 Table). In contrast, the already very low transcript level of *OATP1b3* compared to human liver being 1.2% for HepaRG-Static, was 2-fold further decreased by DMF-culturing. On the other hand, the transcript level of the efflux transporter multidrug resistance-associated protein *MRP2*, was 1.4-fold increased with DMF-culturing (Fig 3E, S2 Table).

Unexpectedly, despite the significant increase of the transcript level of *NTCP*, no difference between DMF- and Static-cultures was observed in the taurocholate-uptake function of NTCP (Fig 3F).

### DMF-culturing does not improve the polarization of the HepaRG cells

**Fig 4:**
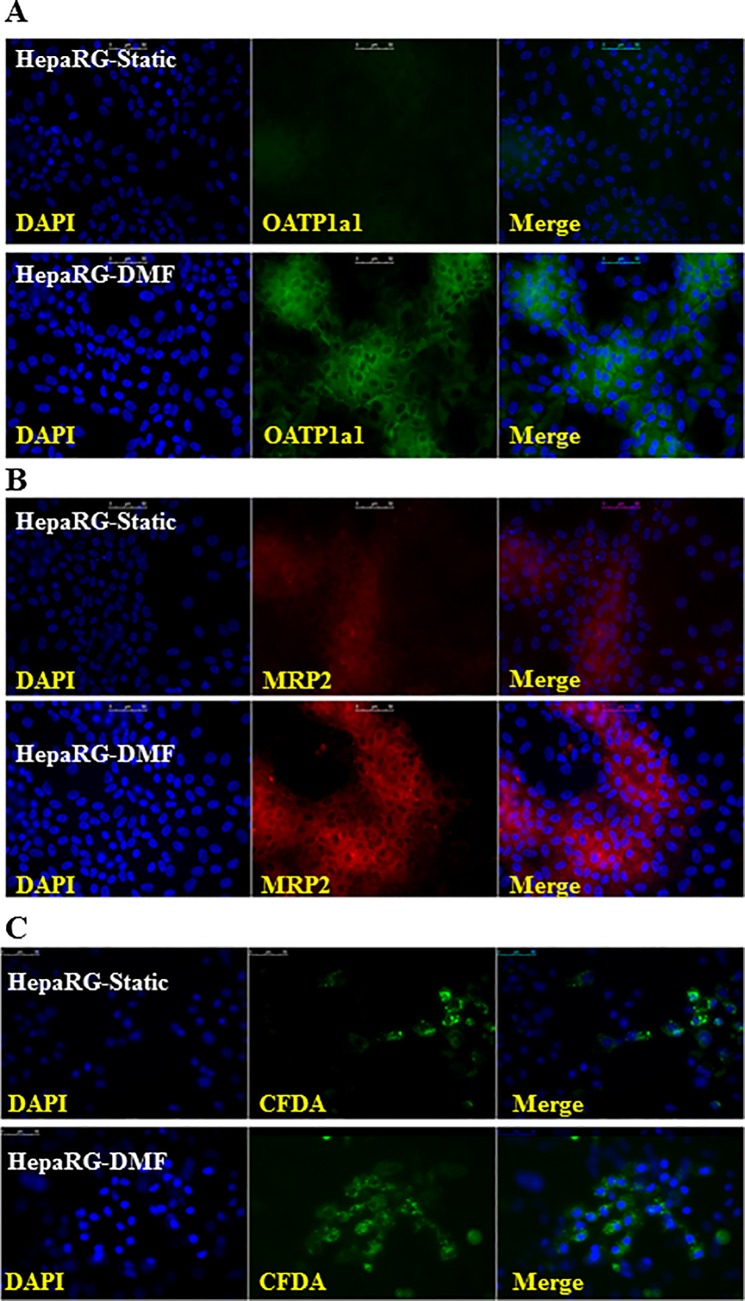
DMF-culturing does not improve the polarization of the HepaRG cells. **(A-B)** Staining of hepatic transporters OATP1a1(green) and MRP2 (red) with DAPI counter-staining for the nuclei (blue). **(C)** Visualization of CFDA (green) with DAPI counter-staining of the nuclei (blue), scale bar = 50μm.

One of the important factors determining transporter function, is the polarization of the hepatocytes, resulting into distinct luminal and basolateral domains that execute the import and export, for *e*.*g*. proteins and bile acid [30]. The polarization of Static- and DMF-cultures was studied by immune-staining of OAPT1a1 and MRP2. The expression of OATP1a1 and MRP2 was increased in the DMF-cultures (Fig 4A and 4B). However, the localization of these hepatic transporters was predominantly cytosolic with minimal membrane localization, indicating that the polarization of the HepaRG cells was absent in both Static- and DMF-cultures. The disturbed polarization of the hepatic transporters was further confirmed by CFDA polarization assay. CFDA, upon entering hepatocytes, is converted into green fluorescent carboxyfluorescein (CF) by intracellular esterases, which then is effluxed to the canalicular side mainly through MRP2. Although CF-signal was increased in the DMF-cultures compared to Static-cultures, no clear canalicular localization was observed under both conditions, confirming that the polarization of the HepaRG cells was not yet established (Fig 4C).

### DMF-culturing induces a shift in cellular energy metabolism towards more mitochondrial-dependent in HepaRG cells

**Fig 5:**
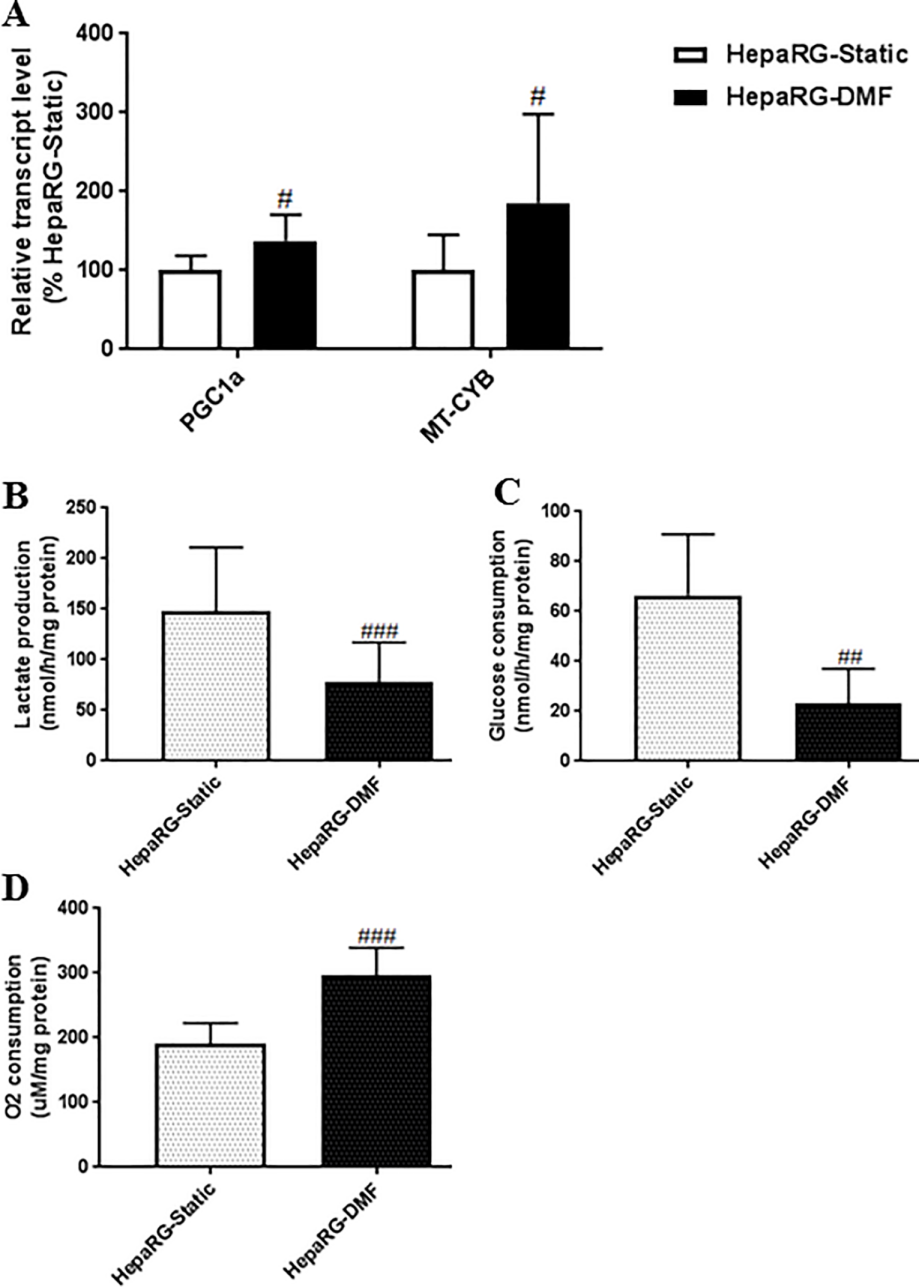
DMF increases mitochondrial energy metabolism of HepaRG cells. **(A)** Transcript levels of *PGC1a* and *MT-CYB*, n = 6/group. **(B-D)** Lactate production, glucose and oxygen consumption, n = 9-15/group. Significance Static *vs* DMF: # = *P* value <0.05, ## = *P* value <0.01, ### = *P* value <0.001 and #### = *P* value <0.0001.

Previously, we found that culturing of HepaRG cells under DMF increased mitochondrial abundance and OxPhos complexes [11]. Here, we further assessed cellular-energy metabolism and mitochondrial functions. In agreement with the previous findings, the transcript level of peroxisome proliferator-activated receptor gamma coactivator 1-alpha (*PGC1a*), the master regulator of mitochondrial biogenesis, was 1.4-fold increased under DMF (Fig 5A, S2 Table). Moreover, the transcript levels of mitochondrially-encoded cytochrome B gene (*MT-CYB*) was 1.7-fold enhanced (Fig 5A, S2 Table). Furthermore, lactate production and glucose consumption were 1.9- and 2.5-fold reduced, whereas oxygen consumption was 1.6-fold increased in DMF-HepaRG *vs* HepaRG-Static cultures (Fig 5B–5D). Collectively, these data confirm that DMF-culturing enhances mitochondrial functions and exerts a shift in cellular-energy metabolism towards OxPhos in HepaRG cells.

### DMF-culturing substantially stimulates hepatic functions and mitochondrial metabolism of C3A cells

**Fig 6:**
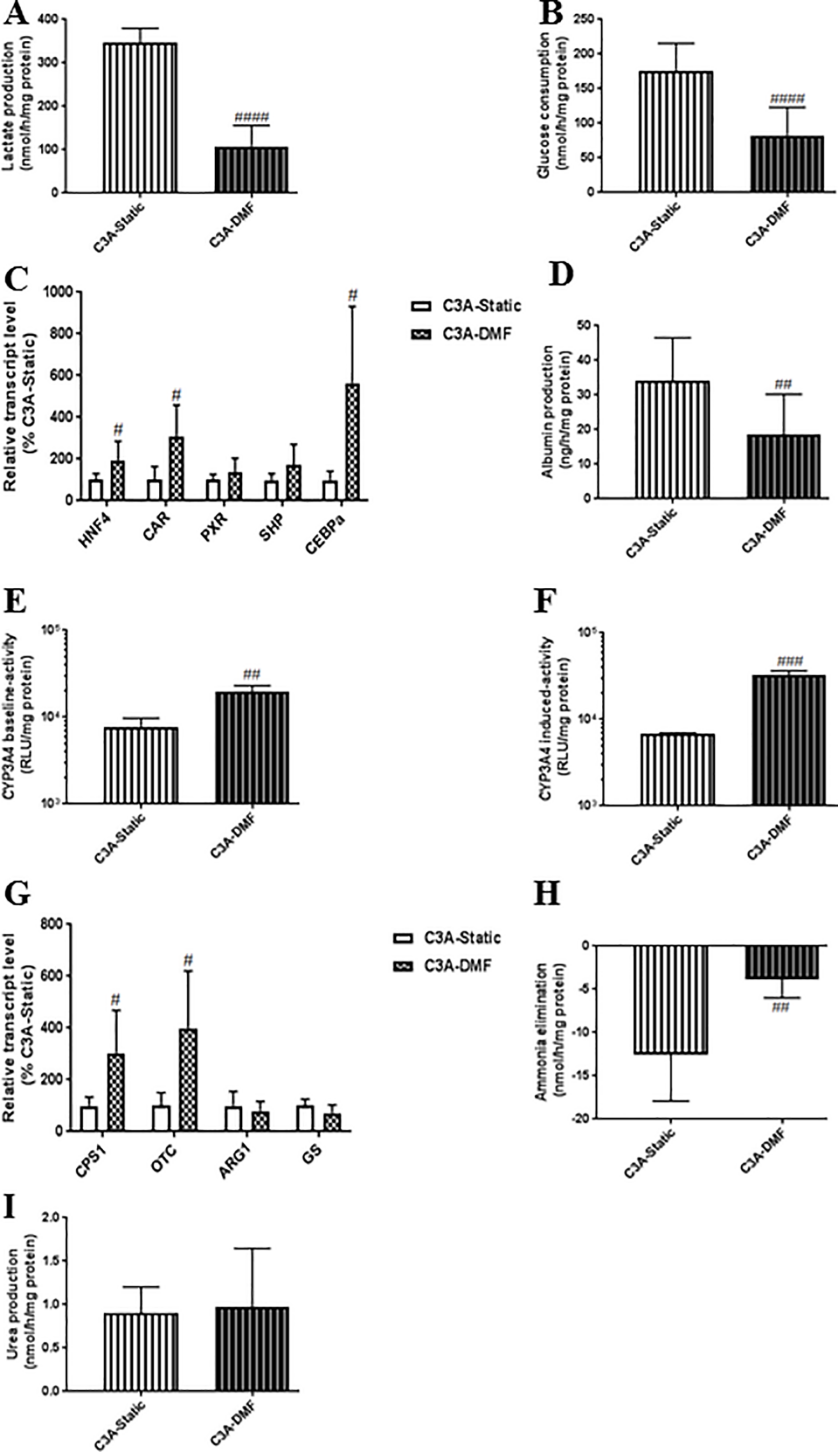
DMF-culturing enhances mitochondrial energy metabolism and hepatic functions of C3A cells. **(A-B)** Lactate production and glucose consumption, n = 6/group. **(C)** Transcript levels of genes encoding hepatic differentiation regulators, n = 6/group. (D) Albumin synthesis. **(E-F)** CYP3A4 activity, n = 3/group. **(G)** Transcript levels of genes involved in nitrogen metabolism, n = 6/group. **(H-I)** Ammonia elimination and urea production, n = 3-6/group. Significance Static *vs* DMF: # = *P* value <0.05, ## = *P* value <0.01, ### = *P* value <0.001 and #### = *P* value <0.0001.

To assess whether the effect of DMF-culturing is cell line (HepaRG) specific, we also compared Static and DMF-cultures of the C3A cell line. Compared to C3A-Static, C3A-DMF relied more on mitochondrial energy metabolism, as indicated by a 3.2- and 2.3-fold reduced lactate production and glucose consumption, resp. (Fig 6A and 6B). In addition, DMF-culturing increased hepatic differentiation, as indicated by a 1.9-,3.1- and 5.6-fold induction of the transcript levels of *HNF4*, *CAR* and *CEBPα*, while *PXR* and *SHP* exhibited a positive trend (Fig 6C, S3 Table). Unexpectedly, Albumin synthesis was reduced in C3A-DMF cultures compared to C3A-Static (Fig 6D, S4 Table). On the other hand, the baseline and induced-CYP3A4 activity was 2.5- and 4.7- fold increased by DMF-culturing (Fig 6E and 6F). In contrast to HepaRG cells, C3A cells produce ammonia and are not capable of eliminating ammonia. C3A cells maintained under DMF-cultures displayed an improved ammonia metabolism, as shown by induction of the transcript levels of the genes encoding urea cycle enzymes *CPS1* and *OTC*, 3.0- and 4.0-fold, resp., whereas the levels of *ARG1* and *GS* remained comparable to that of Static-cultures (Fig 6G, S3 Table). Accordingly, ammonia metabolism was positively modulated, resulting in a 3.3-fold less ammonia production, while urea production remained unchanged (Fig 6H and 6I).

## Discussion

There is an unmet need for a hepatocyte culture platform that either maintains the hepatic functionality of primary hepatocytes and/or induces hepatic functionality of proliferative sources of hepatocytes, such as stem cells and cell lines. This study presents a practice-changing culture method comprised of simply shaking of monolayer cultures after reaching confluence, which substantially increases the hepatic functionality and shifts energy metabolism towards OxPhos of two hepatocyte cell lines, HepaRG and C3A.

Recently, DMF was found to be one of the factors contributing to the enhanced mitochondrial biogenesis under AMC-BAL-culturing [11]. In this study, we further established that DMF-culturing exerted a clear shift in energy metabolism from aerobic glycolysis towards OxPhos, as indicated by the significant decline in lactate production, and glucose consumption in both HepaRG and C3A cell lines and, only tested in HepaRG cells, an increase in oxygen consumption. Additionally, DMF upregulated hepatic transcription factors and hepatic functions of HepaRG and C3A cells, including ammonia metabolism and CYP3A4 activity. More extended studies, in HepaRG cells, showed that DMF induced the transcript levels of various hepatic genes, bile acid synthesis, whereas the expression of the stem cell marker SOX9 was reduced, and its localization was shifted from nuclear towards cytoplasmic, indicating a higher degree of hepatic differentiation under DMF. Therefore, we established a simple modification to conventional culturing methods that upregulates mitochondrial energy metabolism and hepatic differentiation of liver cell lines.

Solid association between mitochondrial energy metabolism and differentiation has recently been established, particularly in stem cell research. Undifferentiated stem cells retain pluripotency and high proliferative potentials under glycolysis-stimulating conditions [31, 32]. Furthermore, reverse mitochondrial remodeling occurs during reprogramming of somatic cells into induced pluripotent stem cells (iPSCs) and during dedifferentiation of primary hepatocytes [16]. Conversely, differentiation of stem cells is associated with mitochondrial remodeling and a shift of energy metabolism towards OxPhos [16, 33].

Although, the hepatic functions of the C3A cells were significantly increased under DMF-culturing, yet ammonia production was not converted into elimination rendering the usage of this cell line as a biocomponent in BALs to support patients with liver failure, questionable. Importantly, the effect of DMF-culturing on the hepatic differentiation of different liver cell lines may vary in its magnitude dependant on the basal hepatic differentiation of the specific cell line under static conditions.

The importance of DMF for HepaRG cells was already shown in the AMC-BAL culture platform [34]. A medium flow rate of 5 ml/min optimally stimulated hepatic functions, whereas lower and higher flow rates resulted in decreased functionality and increased cell damage, resp. Here, we mimicked DMF of the BAL system in monolayers using a simple technique by continuously shaking the cultures at 60 rpm rate during the differentiation phase. Lower shaking rates, being 5 and 25 rpm, were not effective (S3 Fig). In addition, DMF of less dense (sub-confluent) cultures was detrimental to the cells; DMF starting at 3 days after seeding, instead of 14 days, lead to >80% cell death, as observed microscopically.

DMF has also been applied for hepatocyte culturing by others, relying on complex systems, such as microfluidic chambers [35–38]. Importantly, unlike DMF by simple shaking applied in our study, in all above mentioned DMF platforms, an internal oxygenation system was crucial for the viability and functionality of the hepatocytes in agreement with the assumption that improved oxygenation under shaking, stimulates mitochondrial biogenesis and thereby hepatic differentiation. Consistent with this, we recently established that oxygenation with 40%O_2_ instead of 20%O_2_ increased mitochondrial biogenesis, and hepatic differentiation of HepaRG cells Static-monolayers, however not to the level of DMF-culturing [11, 39], suggesting a shared underlying mechanism, relying, at least in part, on improved oxygenation, however this requires further investigations.

Interestingly, culturing of the HepaRG cells under DMF increased their hepatic differentiation superior to DMSO-treatment, without inducing any cytotoxic effect, as found for DMSO-treatment [3, 6]. Ammonia metabolism and the transcript levels of several hepatic genes (*e*.*g*. *HNF4*, *PXR*, *CAR* and *CYP3A4*) were relatively high in the HepaRG-DMF cultures, while only the transcript level of *CYP2B6* among all tested hepatic genes, was more upregulated by DMSO-treatment *vs*. DMF culturing.

The DMF-induced gain of hepatic functionality is probably mediated through upregulation of hepatogenic transcription factors. DMF increased the transcript levels of *HNF4*, *CEBPa* and *CAR* in HepaRG and C3A cells, whereas *PXR* and *SHP*, were induced in HepaRG cells. SHP mediates FXR signaling as a small heterodimer partner. Among other functions, FXR acts as a transcriptional regulator of bile acid biosynthesis and transport in liver [40]. Interestingly, Godoy *et al*. found that three of these regulators, *i*.*e*. *CAR*, *FXR* and *PXR*, were, together with *HNF1*, strongly repressed in cultivated hepatocytes, suggesting that the current *in vitro* culturing models lack stimuli required to maintain gene expression of these regulators in hepatocytes [41], which can be counteracted by DMF-culturing. The upregulation of *HNF4* and *CEBPa* will also contribute to hepatic functionality, as these two transcription factors play a major role in governing hepatic development and regulating a number of critical metabolic pathways, including the urea cycle [42, 43]. CEBP*a* overexpression in combination with HNF4 and FOX2a in adult-liver derived progenitor cells resulted in increased hepatic differentiation [44].

Of note, in HepaRG cells, the production of bile acids, being versatile signaling molecules, was 2.6-fold augmented by DMF-culturing, which may further upregulate FXR, CAR, PXR and other relevant pathways, as cAMP synthesis, and protein kinase C activation [45]. Interestingly, DMF-culturing did not increase albumin synthesis in HepaRG cells and resulted in a reduced albumin production by C3A cells. This unexpected observation might be related to the fact that albumin production is already higher or comparable to the level of human primary hepatocytes in HepaRG and C3A cells cultures resp and could not be further increased by DMF-culturing.

The expression of several hepatic transporters, that were relatively low expressed in HepaRG-Static monolayers, such as NTCP, was increased in HepaRG-DMF cultures, however probably due to limited polarization, NTCP function remained unchanged. Hepatocyte polarization is a complex process, leading to an organized localization of extracellular, cytoskeletal and tight junction molecules [46]. The polarization is more established in HepaRG monolayers when treated with 1.7% DMSO for the last two weeks of culturing [47]. In the past increased polarization of HepaRG cells was achieved by AMC-BAL culturing of HepaRG cells [26]. Therefore, new culture techniques combining DMF by simple shaking and 3D configuration may thus represent a potential window for further improving *in vitro* hepatocyte culturing.

## Conclusions

The current study demonstrates that DMF-culturing substantially enhances mitochondrial energy metabolism and hepatic functions in two different human liver cell lines. These findings strongly support a role of mitochondria in regulating the differentiation of human liver cells. Importantly, we developed a new easily applicable and scalable culture platform that substantially enhances the functionality of human liver cell lines. Considering the high similarity of responsiveness to DMF-culturing between two hepatic cell lines, it is likely that DMF will also exert positive effects on hepatic differentiation of stem cells and maintenance of differentiation of primary hepatocytes.

## Supporting information

S1 Table Primers used in the RT-qPCR and amplicon size. (DOC)

S2 Table Transcript levels of genes in HepaRG cultures as a % of human livers. (DOC)

S3 Table Transcript levels of genes in C3A cultures as a % of human livers. (DOC)

S4 Table Hepatic functions of primary human hepatocytes (PHHs), HepaRG and C3A static and DMF-cultures. Values are given as mean±SD, for more details [6, 48].
(DOC)

S1 Fig Higher resolution staining of albumin (green), and SOX9 (red), with DAPI counter-staining of the nuclei (blue) in HepaRG-Static, HepaRG-DMSO-Static and HepaRG-DMF cultures. The arrow indicates the nuclear translocation of SOX9 in HepaRG-Static, scale bar = 50μm.
(TIF)

S2 Fig Higher resolution staining for CEBPα (red), with DAPI counter-staining of the nuclei (blue) in HepaRG-Static, HepaRG-DMSO-Static and HepaRG-DMF cultures. The arrow indicates nuclear translocation of CEBPα, observed in HepaRG-DMF, scale bar = 50μm.
(TIF)

S3 Fig Optimization of the shaking rate for DMF-cultures. Briefly, HepaRG monolayers were kept statically for two weeks (the proliferation phase), then cultures were moved to a shaking incubator with 5, 25 or 60 rpm during the differentiation phase (the last two weeks of culturing). Hepatic functionality was evaluated for ammonia elimination, urea production and lactate production, of different DMF-cultures, compared to HepaRG-Static cultures.
(TIF)

S1 Data Excel sheet with all raw data. (XLS)
