# Supplementary material for: A practice-changing culture method relying on shaking substantially increases mitochondrial energy metabolism and functionality of human liver cell lines
Source: PLoS One. 2018 Apr 19;13(4):e0193664. doi: 10.1371/journal.pone.0193664 (PMC5908182; doi:10.1371/journal.pone.0193664)
Supplement: S1 Table — (DOC) [file pone.0193664.s001.doc]

**“S1 Table.” Primers** used in the RT-qPCR and amplicon size.

| Gene | Sense sequence | Anti-sense sequence | bp |
| --- | --- | --- | --- |
| *18S* | TTCGGAACTGAGGCCATGAT | CGAACCTCCGACTTTCGTTCT | 151 |
| *ARG1* | TTGGCAAGGTGATGGAAGAAACA | CCTCCCGAGCAAGTCCGAAACAA | 305 |
| *ARG2* | GGTCCCGCTGCCATAAGAGA | GGCATCAACCCAGACAACACAA | 299 |
| *CAR* | CGTCATGGCCAGTAGGGAAG | CATGCCAGCATCTAAGCACT | 232 |
| *CEBPa* | CGGCGGCGGCGACTTTGA | GGCGGCGGCGGCTGGTA | 254 |
| *CPS1* | GAAGGGGCCCGAGAAGTAGAA | CTCAACCGGGGCCAGGAAAAC | 445 |
| *CYP3A4* | CATAGCCCAGCAAAGAGCAAC | GACCATCATAAAAGCCCCACAC | 313 |
| *CYP2B6* | CCCGCCCTCTGCCCCTTTTG | TCCACACTCCGCTTTCCCATCC | 328 |
| *GS* | GCCTGCTTGTATGCTGGAGTC | GGCGCTACGATTGGCTACAC | 420 |
| *HNF4* | TCCGGGCTGGCATGAAGAAGG | CCAGGGGGAGCTCGCAGAAAG | 321 |
| *MT-CYB* | AACTTCGGCTCACTCCTTGG | CCCGATGTGTAGGAAGAGGC | 204 |
| *MRP2* | AGCACCGACTATCCAGCATCTC | ATCCGGCCTGTGGGTGTTGTG | 205 |
| *NTCP* | GGCTTTCTGCTGGGTTATGTT | GGGGAAAGAAGAAAAGTGGTC | 162 |
| *OATP1b1* | TACCCTGGGATCTCTGTTTTCTAA | GGCTGACCATACTGTTGCTCTAC | 437 |
| *OATP1b3* | GCACACTTGGGTGAATGCCCA | AAGCTCCTTGTGCTCCACAGC | 283 |
| *OTC* | GCCGGATGCTAGTGTAACCAA | AGCCGCTTTTTCTTCTCCTCTTC | 161 |
| *PGC1a* | CAGGTGCCTTCAGTTCACTCT | AACCAGAGCAGCACACTCGAT | 181 |
| *POR* | ATTCGCCAGTACGAGCTTGT | AGTCCGAGATGTCCAATTCC | 205 |
| *PXR* | CGCCTGCGCAAGTGCCTGGAG | GTCGGCTGGGGGTTTGTAGTTC | 420 |
| *SHP* | CGCCCTATCATTGGAGATGT | TGTCTATACAGGCTTGCCCC | 175 |
| *UGT1a1* | CCCATTCTCCTACGTGCCCAGG | GGTTCCAGTGTACCGCCACAGG | 457 |
