# Supplementary material for: A practice-changing culture method relying on shaking substantially increases mitochondrial energy metabolism and functionality of human liver cell lines
Source: PLoS One. 2018 Apr 19;13(4):e0193664. doi: 10.1371/journal.pone.0193664 (PMC5908182; doi:10.1371/journal.pone.0193664)
Supplement: S2 Table — (DOC) [file pone.0193664.s002.doc]

**“S2 Table.” Transcript levels of genes in HepaRG cultures as a % of human livers.**

| **Gene** | **HepaRG-Static** | **HepaRG-DMF** | ***P* value**  **Static *vs* DMF** |
| --- | --- | --- | --- |
| *ARG1* | 2.7 ± 0.9 | 16.2 ± 6.1 | 0.000 |
| *ARG2* | 1406.8 ± 952.0 | 1413.5 ± 308.7 | 0.987 |
| *CAR* | 2.2 ± 1.5 | 4.4 ± 2.2 | 0.000 |
| *CEBPa* | 17.1 ± 8.4 | 41.1 ± 13.2 | 0.000 |
| *CPS1* | 30.3 ± 19.1 | 56.0 ± 20.7 | 0.049 |
| *CYP3A4* | 2.3 ± 1.0 | 33.7 ± 19.6 | 0.003 |
| *CYP2B6* | 4.5 ± 3.7 | 11.1± 6.1 | 0.047 |
| *GS* | 486.5 ± 154.7 | 800.4 ± 231.2 | 0.030 |
| *HNF4* | 242.5 ± 62.5 | 351.6 ± 74.2 | 0.020 |
| *MT-CYB* | 58.2 ± 26.3 | 108.0 ± 65.5 | 0.031 |
| *MRP2* | 64.9 ± 20.4 | 83.4 ± 20.3 | 0.001 |
| *NTCP* | 16.4 ± 13.4 | 36.7 ± 24.1 | 0.031 |
| *OATP1b1* | 3.2 ± 2.5 | 7.1 ± 5.4 | 0.005 |
| *OATP1b3* | 1.2 ± 0.7 | 0.5 ± 0.3 | 0.006 |
| *PGC1a* | 462.9 ± 257 | 573.6 ± 361.9 | 0.035 |
| *POR* | 40.5 ± 21.7 | 58.4 ± 24.7 | 0.002 |
| *PXR* | 9.7 ± 5.7 | 16.5 ± 6.3 | 0.000 |
| *SHP* | 11.4 ± 5.1 | 20.2 ± 7.1 | 0.040 |
| *UGT1a1* | 46.6 ± 17.1 | 74.2 ± 21.6 | 0.006 |
