# Supplementary material for: A practice-changing culture method relying on shaking substantially increases mitochondrial energy metabolism and functionality of human liver cell lines
Source: PLoS One. 2018 Apr 19;13(4):e0193664. doi: 10.1371/journal.pone.0193664 (PMC5908182; doi:10.1371/journal.pone.0193664)
Supplement: S3 Table — (DOC) [file pone.0193664.s003.doc]

**“S3 Table.” Transcript levels of genes in C3A cultures as a % of human livers.**

| **ID** | **C3A-Static** | **C3A-DMF** | ***P* value**  **Static *vs* DMF** |
| --- | --- | --- | --- |
| *ARG1* | 0.2 ± 0.1 | 0.2 ± 0.1 | 0.597 |
| *CAR* | 0.8 ± 0.5 | 2.4 ± 1.2 | 0.029 |
| *CEBPa* | 15.2±6.4 | 85.6±55.6 | 0.011 |
| *CPS1* | 2.9 ± 1.0 | 8.7 ± 4.8 | 0.039 |
| *GS* | 406.3 ± 100.0 | 289.4 ± 131.4 | 0.188 |
| *HNF4* | 1.3 ± 0.4 | 2.5 ± 1.2 | 0.039 |
| *OTC* | 0.4 ± 0.2 | 1.6 ± 0.9 | 0.029 |
| *PXR* | 20.1 ± 5.5 | 27.6 ± 13.4 | 0.321 |
| *SHP* | 215.6 ± 65.3 | 368.1 ± 213.6 | 0.194 |
