# Supplementary material for: A practice-changing culture method relying on shaking substantially increases mitochondrial energy metabolism and functionality of human liver cell lines
Source: PLoS One. 2018 Apr 19;13(4):e0193664. doi: 10.1371/journal.pone.0193664 (PMC5908182; doi:10.1371/journal.pone.0193664)
Supplement: S4 Table — Values are given as mean±SD, for more details [6, 48]. (DOC) [file pone.0193664.s004.doc]

**“S4 Table.” Hepatic functions of primary human hepatocytes (PHHs), HepaRG and C3A static and DMF-cultures. Values are given as mean±SD, for more details .**

| **Hepatic function** | **HepaRG-Static** | **HepaRG-DMF** | **C3A-Static** | **C3A-DMF** | **PHHs** |
| --- | --- | --- | --- | --- | --- |
| Ammonia elimination  (nmol/h/mg protein) | 30+10.5**** | 59.6±22.4 ^NS^ | -12.5±5.4**** | -3.8±2.2*** | 95±5.0 |
| Urea production  (nmol/h/mg protein) | 1.7±0.9 **** | 4.1±1.3 **** | 0.9±0.3 ** | 1.0±0.7 ** | 91±34 |
| Bile acid production  (nmol/h/mg protein) | 0.1±0.0**** | 0.18±0.0**** | ND | ND | 1.5±1.1 |
| Albumin production  (ng/h/mg protein) | 51.5±10.3** | 48.4±5.3* | 34.2±12.3 ^NS^ | 18.6±11.6** | 37.7±7.8 |

**ND**: not determined

**NS**: not significant

*****: indicates significance *vs* PHHs
