# Supplementary figures and images for: A practice-changing culture method relying on shaking substantially increases mitochondrial energy metabolism and functionality of human liver cell lines
Source: PLoS One. 2018 Apr 19;13(4):e0193664. doi: 10.1371/journal.pone.0193664 (PMC5908182; doi:10.1371/journal.pone.0193664)

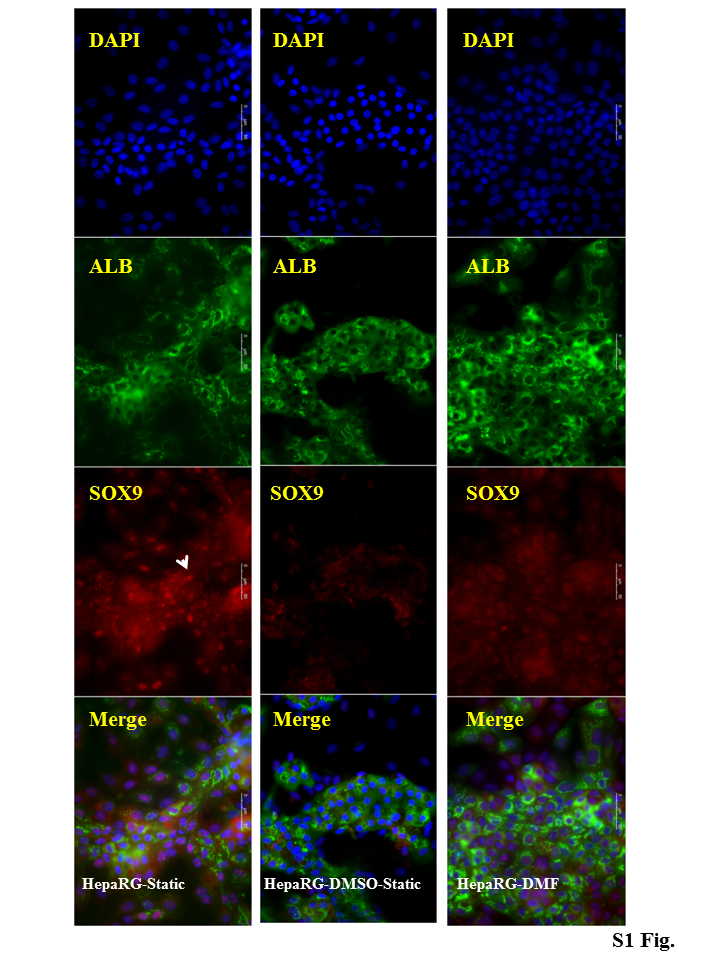

Supplement: S1 Fig — The arrow indicates the nuclear translocation of SOX9 in HepaRG-Static, scale bar = 50μm. (TIF) [file pone.0193664.s005.tif]

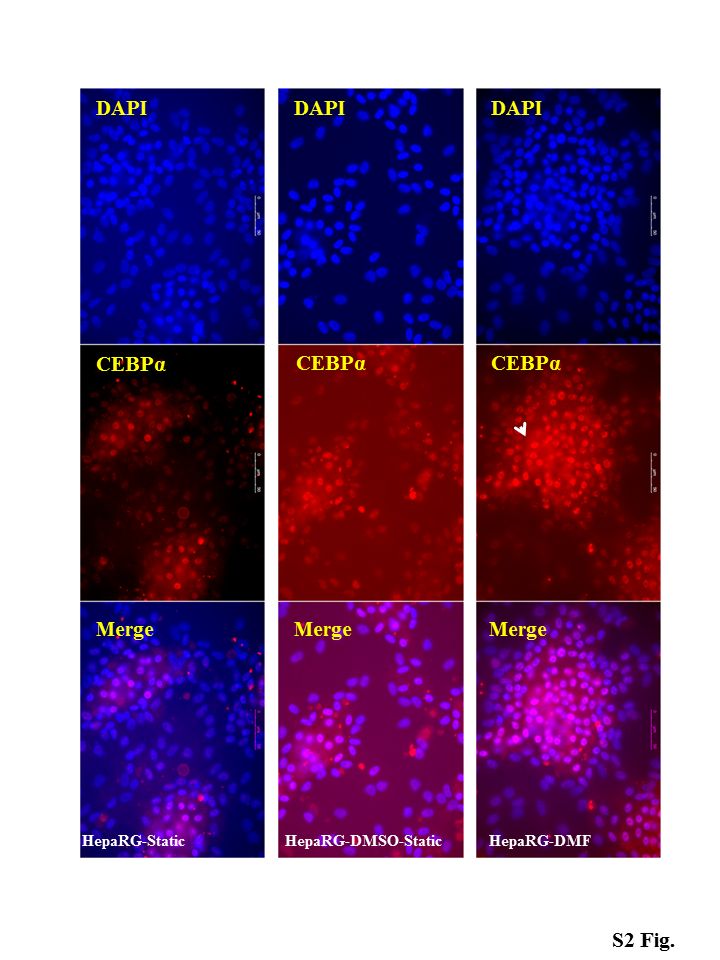

Supplement: S2 Fig — The arrow indicates nuclear translocation of CEBPα, observed in HepaRG-DMF, scale bar = 50μm. (TIF) [file pone.0193664.s006.tif]

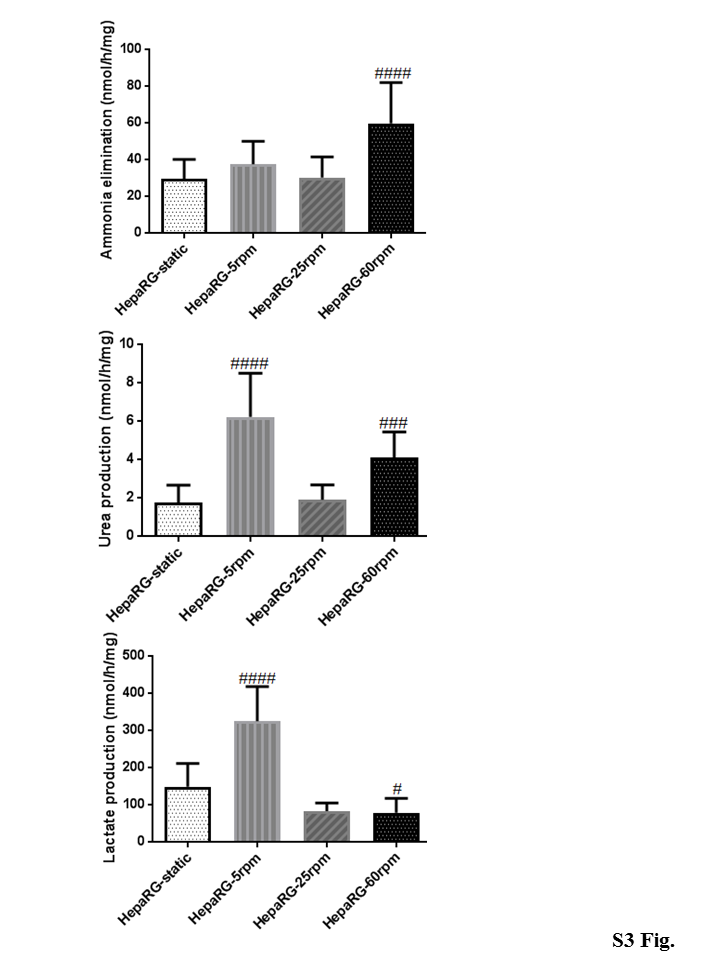

Supplement: S3 Fig — Briefly, HepaRG monolayers were kept statically for two weeks (the proliferation phase), then cultures were moved to a shaking incubator with 5, 25 or 60 rpm during the differentiation phase (the last two weeks of culturing). Hepatic functionality was evaluated for ammonia elimination, urea production and lactate production, of different DMF-cultures, compared to HepaRG-Static cultures. (TIF) [file pone.0193664.s007.tif]
